# Students’ Perceptions of Online Learning and their Correlations with Deep Learning and Proactive Decision-making in Higher Education

**DOI:** 10.12688/f1000research.178825.2

**Published:** 2026-10-26

**Authors:** Venny Rosalina, Yenni Rozimela, Indrayuda Indrayuda, Fabio Yuda, Erwin Mardiansyah

**Affiliations:** 1 Dance Education, Universitas Negeri Padang, Padang, West Sumatra, 25131, Indonesia; 2 English Language Education, Universitas Negeri Padang, Padang, West Sumatra, 25131, Indonesia; 3 Education in Drama, Dance, and Music Arts, Universitas Negeri Padang, Padang, West Sumatra, 25131, Indonesia; 4 Arts, Institut Seni Indonesia Surakarta, Surakarta, Central Java, 57126, Indonesia; 5 Dance Arts, Institut Seni Budaya Indonesia Bandung, Bandung, West Java, 40265, Indonesia

**Keywords:** Deep learning, learning autonomy, online learning, proactive decision-making, quality of online learning

## Abstract

Online learning increases student flexibility and engagement, but its effectiveness depends on a design that encourages deep learning and proactive decision-making rather than just surface activities. This study aims to examine the relationships among student perceptions of online learning, deep learning tendencies, and proactive decision-making abilities in 202 students (M ± SD = 20.46 ± 1.75). Data were collected using the Distance Education Learning Environments Survey (DELES), the Revised Two Factor Study Process Questionnaire (R-SPQ-2F), and the Students’ Proactive Decision-Making Scale (SPDMS-18). Spearman’s test showed that positive perceptions of online learning were significantly related to deep learning (ρ = 0.634, p < 0.001) and proactive decision-making (ρ = 0.474, p < 0.001). Descriptive results showed a positive trend in all online learning indicators, fluctuations in motivation and deep learning strategies in the middle semester, and high stability in the proactive decision-making aspect. These findings confirm that high-quality online learning experiences encourage the adoption of deep learning approaches and proactive decision-making, including intrinsic motivation, analytical strategies, initiative, and goal-setting. Practical implications emphasize the importance of digital pedagogy training for lecturers, the development of e-learning systems that support student independence, and curricula that stimulate analysis and reflection. This research contributes to the design of digital-based higher education oriented toward 21st-century competencies.

## Introduction

In today’s modern education era, where information and knowledge rapidly evolve on digital platforms, deep learning and proactive decision-making are crucial aspects of student competency development. Deep learning focuses on memorizing information and knowledge, conceptual understanding, critical application of knowledge, analytical thinking and problem-solving, creativity, and collaboration ( Hattie & Donoghue, 2016; Zebua, 2025). These learning experiences provide students with ample opportunities to design their learning goals and develop a deeper understanding and meaning of the learning process ( Biggs & Tang, 2011; Kovač et al., 2025). With the increasing prevalence of student-centered learning approaches and the use of technology, students must become active learners capable of exploring, analyzing, and synthesizing information independently and reflectively without having to rely heavily on others (such as lecturers).

Meanwhile, proactive decision-making teaches students to take the initiative in planning and choosing learning strategies, considering future events when making current decisions. They project it before it becomes a crisis ( Blegur et al., 2025). Proactive students do not wait for instructions but take an active role in managing their learning ( Siebert et al., 2021), which has been shown to increase their career calling and academic motivation ( Frolova & Mahmood, 2025), while also encouraging students’ knowledge-sharing behavior through mediating learning engagement and a positive classroom climate ( Lin et al., 2024). In the context of distance learning or online learning, these two variables are increasingly important. Without the physical presence of lecturers, students must be able to regulate themselves to achieve meaningful learning and make independent learning decisions. That is why universities need to consolidate learning environments that encourage deep understanding while facilitating their students’ proactive decision-making, rather than convincing students that lecturers are the center of information and knowledge.

Although deep learning and proactive decision-making are strategic goals in higher education, many students struggle with both aspects. The main problem with deep learning is the dominance of a surface learning approach, where students tend to memorize material to pass exams without understanding its deeper meaning, hindering the development of higher-order cognitive skills and the capacity to solve real-world problems ( Wang, 2024). However, intensive study preparation just before exams does not predict student performance ( von Keyserlingk et al., 2025). High assignment loads, academic pressure, and lecturer-centered teaching methods drive it. A research report by Lindblom-Ylänne et al. (2019) clearly recorded the percentage of elements associated with surface learning: lack of organized learning (48%), low learning motivation (31%), weak self-efficacy (30%), lack of self-regulation (25%), negative learning experiences (25%), low interest in learning (20%), and heavy workloads (7%).

Students’ proactive decision-making is also often underdeveloped. Many students are still reactive, only studying when exams are approaching or given instructions ( Al-Yousifi & Murad, 2022; Blegur, 2025; Blegur et al., 2025). Lack of self-regulation skills, analytical thinking, goal-setting, and proactive decision-making, resulting in reliance on lecturers’ guidance, are among the inhibiting factors ( Blegur et al., 2021, 2023; Frolova & Mahmood, 2025; Li et al., 2025). In online learning, these issues are further complicated by the lack of direct interaction and external control. Students not accustomed to analytical and critical thinking and making independent learning decisions will struggle to navigate the online learning process in depth and meaningfully. If left unaddressed, this phenomenon can hinder academic achievement, reduce learning satisfaction, and worsen the quality of graduate competencies. Therefore, pedagogical approaches and affirmative learning environments need to be designed to foster these two important skills comprehensively and sustainably.

Research on online learning has consistently demonstrated its effectiveness in educational contexts. H.-C. Liu and Yen (2014) found that online learning positively impacted learning effectiveness, particularly in curriculum management, instruction, and technological media. A meta-analysis by Martin et al. (2021) also showed that synchronous online learning had a small but significant effect on cognitive outcomes. This effectiveness was moderated by course duration, teaching method, and learner level. Kuo et al. (2023) emphasized the importance of collaborative learning in online learning, as this approach increases motivation, self-efficacy, and reflection, while reducing cognitive load. Similarly, AL-Momani et al. (2024) successfully reported the impact of online learning on university educational outcomes, with average satisfaction scores indicating student consensus. More recently, Nusantara et al. (2024) added that although online learning positively impacted student achievement, the difference was insignificant compared to conventional learning, depending on the domain, educational level, and subject.

Previous studies have shown that online learning positively impacts various aspects of education, such as instructional effectiveness, technological media, and basic cognitive outcomes. However, most research still focuses on superficial learning outcomes such as satisfaction and efficiency. Few studies have examined how online learning can foster deep learning, critical thinking skills, reflective thinking, comprehensive conceptual understanding, and meaningful integration of knowledge. Furthermore, the relationship between online learning and students’ proactive decision-making has not been adequately explored. Proactive decision-making supports independent and lifelong learning in the digital and independent learning era. Finally, the novelty of this research lies in its attempt to examine online learning that not only focuses on basic cognitive aspects but also facilitates students’ deep learning and proactive decision-making.

## Literature review

### Online learning and deep learning

Online learning has become a common method adopted by higher education institutions worldwide. However, the success of online learning implementation is not only measured by technical aspects or academic achievement, but also by the extent to which this approach can foster deep learning in students, strengthening online learning in the modern education era. Deep learning builds high-level competencies, namely critical thinking, problem-solving, and innovation, which are essential for innovative talent in the 21st century ( Weng et al., 2023) through a process of inquiry and building new relationships with and among students, families, and communities ( Fullan et al., 2018). The primary goal of deep learning is to help students acquire competencies and dispositions that can prepare them to become creative, connected, and collaborative problem solvers for life, as well as healthy and holistic individuals who not only contribute to but also create the common good in the world ( Fullan & Langworthy, 2014). Thus, deep learning is a sustainable mindset by connecting learning content with students’ intelligence, emotions, and life values ( Kovač et al., 2025).

According to Çebi et al. (2023), online learning allows students to access learning content and record their interactions freely. This experience gives students the flexibility to control their learning time, approach learning at their own pace, and play a key role in deciding on learning strategies ( Akpen et al., 2024). This autonomy is crucial in supporting deep learning because it allows students to solve problems, make their own learning decisions, and explore topics based on their interests and goals. Well-designed online learning models, such as project-based learning, help students improve their analytical, critical, and creative thinking skills and enable them to solve problems independently by developing learning strategies tailored to their needs ( Amin & Shahnaz, 2023; Syawaludin et al., 2022). A research report by Zhou et al. (2024) also demonstrated that interactive online instruction using Ryan and Deci (2000) Self-Determination Theory framework significantly impacted deep learning in Chinese students.

Research by Musthofa et al. (2023) demonstrated a positive influence of online learning on students’ professional competence. Specifically, the psychomotor aspect had a 45.7% impact, the affective aspect 44.2%, and the cognitive aspect 37.6%. Furthermore, online learning allows students to see the direct relevance of what they learn to life outside of campus, such as studying real-life cases to understand the material thoroughly. Fullan and Langworthy (2014) also identified students’ previous online learning experiences as an important indicator of deep learning, specifically the ability to relate learning material and problem-solving to the real world. Furthermore, online learning allows students to explore topics in greater depth through flexible content presentation, access to supplementary materials, discussion forums, and independent reflection. It means online learning also incorporates cognitive-emotional experiences and meta-cognitive participation in deep learning (E. Liu et al., 2022). Therefore, online learning is significantly related to students’ deep learning.

### Online learning and students proactive decision-making

In higher education, online learning delivers material and a platform for developing essential 21st-century skills ( Reaves, 2019), including proactive decision-making. These skills reflect students’ ability to take control of their learning process, set goals, evaluate information, and take appropriate action based on critical thinking and careful planning ( Blegur, 2025; Blegur et al., 2025; Siebert et al., 2023). In this regard, online learning can be a significant catalyst for developing proactive decision-making in students if designed and implemented correctly. Consider the research report by Sutiman et al. (2022), which found that online learning has increased optimism and the quality of individual career decision-making by strengthening independence, fostering digital literacy, and monitoring social interactions.

One key indicator of proactive decision-making skills is students’ ability to identify comprehensive and systematic goals ( Blegur, 2025; Blegur et al., 2025; Siebert et al., 2023). Online learning provides a flexible environment and demands high levels of independence, necessitating internal motivation and a strong sense of learning independence ( Sumbawati et al., 2020). This flexible learning environment allows students to set clear and realistic learning goals and manage their time effectively. They must understand a topic and conclude while practicing planning and problem-solving skills. In an online system, students have high accessibility, flexibility, and convenience due to their freedom to choose when and how to learn ( Kamraju et al., 2024), which encourages the development of learning strategies and decisions that align with their academic goals. Examining Crant (2000) proactive behavior theory, its relevance to online learning is that students take the initiative to improve their current situation or create change for the better. They use the flexible online learning environment to diagnose goals, anticipate problems, and create opportunities for success.

Embracing constructivist learning theory, online learning also facilitates students’ independent exploration of information through various interpretive resources to construct their knowledge ( Bada, 2015; Taber, 2024). The advantage of learning in an online environment is the abundance of information sources, thus training students to develop critical thinking by verifying and evaluating information before making academic decisions ( Tathahira, 2020). It includes students developing appropriate learning strategies for their academic needs ( Hongsuchon et al., 2022). Unfortunately, this potential has not been the focus of many researchers. However, the dynamic nature of time, information sources, and learning locations in learning can systematically train students to identify goals, identify alternatives, seek information, use decision radar, take initiative, and strive for improvements in their various academic performance and goals, which are important indicators of proactive decision-making skills ( Blegur, 2025; Blegur et al., 2025; Siebert et al., 2023). Therefore, online learning is a very effective new tool for training students’ proactive decision-making.

## Method

### Design and procedure

A correlational research design was chosen to analyze the relationship between the predictor variable, online learning, and two outcome variables: deep learning and proactive decision-making. This research design aimed to determine the extend to which the implementation of online learning correlates with students’ deep learning and proactive decision-making. Using Spearman’s rank correlation, this approach enabled quantitative analysis of the associations between online learning and students’ cognitive and affective dimensions individually, allowing researchers to evaluate the strength and direction of these relationships for each outcome variable.

This approach is relevant in the current context of digital education, where it is crucial to empirically and systematically understand the effects of online learning on students’ thinking and decision-making. Therefore, we began the study by tracking and establishing data collection instruments for the three research variables and providing instrument items in a Google form in the target language (Indonesian) using a five-point Likert scale. We distributed the online instrument to respondents. The researchers then tabulated the data collected from respondents using Microsoft Excel and SPSS version 29.

### Respondent

The respondents of the study were students of the Dance Education Study Program, Faculty of Languages and Arts, Universitas Negeri Padang, West Sumatra Province, Indonesia. The respondents numbered 202 students (M ± SD = 20.46 ± 1.75), with 12 male students (5.9%) and 190 female students (94.1%) identified using a total sampling technique. They were each spread from Semester I as many as 61 people (30.2%) with 4 males and 57 females, Semester III as many as 45 people (22.3%) with 4 males and 41 females, Semester V as many as 52 people (25.7%) with 3 males and 49 females, and Semester VII as many as 54 people (21.8%) with 1 male and 53 females. The research process has been approved by the ethics committee and was carried out in accordance with the principles of research ethics for students. Before data collection, the researcher provided information on the objectives, procedures, and participants’ rights to participate voluntarily. Respondents are also given the right to refuse or withdraw from participation at any time without consequences.

### Instrument

Respondents’ online learning data were collected using the Distance Education Learning Environments Survey (DELES) developed by Walker and Fraser (2005). This scale (DELES) includes 34 items and has been piloted on 680 people using six indicators with loading factor values between 0.55–0.90. The first indicator, instructor support, consists of 8 items (numbers 1–8) with a reliability value of 0.87, including the item “ *The instructor gives me valuable feedback on my assignments.*” The second indicator, student interaction and collaboration, consists of 6 items (numbers 9–14) with a reliability value of 0.94, including the item “ *I collaborate with other students in the class.*” The third indicator, personal relevance, consists of 7 items (numbers 15–21) with a reliability value of 0.92, including the item “ *I can connect my studies to my activities outside of class.*” The fourth indicator, authentic learning, consists of 5 items (numbers 22–26) with a reliability value of 0.89, including the item “ *I study real cases related to the class.*” The fifth indicator, active learning, consists of 3 items (numbers 27–29) with a reliability value of 0.75, including the item “ *I explore my own strategies for learning.*” The sixth indicator, student autonomy, consists of 5 items (numbers 30–34) with a reliability value of 0.75, including the item “ *I make decisions about my learning.*” Respondents responded on a five-point Likert scale: never-always.

Furthermore, respondents’ deep learning data were collected using the Revised Two Factor Study Process Questionnaire (R-SPQ-2F) developed by Biggs et al. (2001). This scale (R-SPQ-2F) includes 20 items. It has been tested on 724 students using four indicators with a Comparative fit Index value of >0.90, namely deep motive of 0.997, deep strategy of 0.998, surface motive of 0.988, and surface strategy of 0.998, thus indicating good construct validity. The first indicator, deep motive, consists of 5 items (numbers 1, 5, 9, 13, 17) with a reliability value of 0.62, including the item “ *I find that at times studying gives me a feeling of deep personal satisfaction.*” The second indicator, deep strategy, consists of 5 items (numbers 2, 6, 10, 14, 18) with a reliability value of 0.63, including the item “ *I find most new topics interesting and often spend extra time trying to obtain more information about them.*” The third indicator, surface motive, consists of 5 items (numbers 3, 7, 11, 15, 19) with a reliability value of 0.72, including the item “ *I find I can get by in most assessments by memorizing key sections rather than trying to understand them.*” The fourth indicator, surface learning, consists of 5 items (numbers 4, 8, 12, 16, 20) with a reliability value of 0.57, including the item “ *I find the best way to pass examinations is to try to remember answers to likely questions.*” Respondents responded on a five-point Likert scale: never-always.

Finally, the respondents’ proactive decision-making data were collected using the Students’ Proactive Decision-Making Scale (SPDMS-18) developed by Blegur et al. (2025). This scale includes 18 items composed of six indicators, where three items represent each indicator. These have been tested on 849 students with loading factor values between 0.709 and 0.835. The first indicator, systematic identification of objectives (numbers 1–3) with a reliability value of 0.752, includes the item “ *Identifying comprehensive (broad, thorough, meticulous) goals to improve academic performance.*” The second indicator, systematic identification of alternatives (numbers 4–6) with a reliability value of 0.765, includes the item “ *Trying to find new alternatives if the chosen alternatives have not succeeded in achieving the set goals.*” The third indicator, systematic search for information (numbers 7–9), with a reliability value of 0.829, includes the item “ *Verifying the accuracy of information before making decisions.*” The fourth indicator, using a decision radar (numbers 10–12) with a reliability value of 0.75, includes the item “ *Thinking about when to make the right decision based on the situation and condition.*” The fifth indicator, taking initiative (numbers 13–15), with a reliability value of 0.802, includes the item “ *Taking the initiative to ask colleagues and/or lecturers when encountering problems while striving to achieve goals.*” The sixth indicator, striving for improvement (numbers 16–18), with a reliability value of 0.835, includes the item “ *Proactively improving current academic performance to make it better.*” Respondents responded on a five-point Likert scale: disagree-strongly agree.

### Data analysis

The data on online learning, deep learning, and proactive decision-making were then analyzed descriptively and correlatively to examine and test the correlations among these variable. The results of the normality tests using the Kolmogorov-Smirnov and Shapiro-Wilk tests indicates that the online learning variable was normally distributed according to the Kolmogorov-Smirnov test (p = 0.069), although the Shapiro-Wilk test yielded a significant result (p < 0.001). Meanwhile, the deep learning and proactive decision-making variables yielded p-values <0.05 in both tests, indicating that neither variable normally distributed (see Table 1).

**Table 1.** Data normality test.

Variable	Kolmogorov-Smirnov			Shapiro-Wilk		
	Statistic	df	Sig.	Statistic	df	Sig.
Online learning	0.060	202	0.069	0.970	202	<0.001
Deep learning	0.084	202	0.001	0.961	202	<0.001
Proactive decision-making	0.151	202	<0.001	0.909	202	<0.001

Furthermore, the results of the linearity test based on ANOVA show that the significance value of deviation from linearity in the relationship between online learning and deep learning is 0.147 (>0.05), and between online learning and proactive decision-making is 0.290 (>0.05), which indicates that the relationship between variables is linear (see Table 2).

**Table 2.** Data linearity test (ANOVA table).

	Sum of squares	Mean square	F	Sig.
Deep learning * Online learning	7621.702	60	1.246	0.147
Proactive decision-making * Online learning	3784.262	60	1.120	0.290

Because some variables were not normally distributed, Spearman’s correlation was used for statistical testing. If the significance value was <0.05, it was concluded that there was a relationship between the online learning variable and deep learning or proactive decision-making, and vice versa. Correlation coefficients were categorized as follows: 0.00–0.10 (negligible), 0.10–0.39 (weak), 0.40–0.69 (moderate), 0.70–0.89 (strong), and 0.90–1.00 (very strong) ( Dancey & Reidy, 2020; Schober et al., 2018). All data collection and analysis were conducted using Google Forms, Microsoft Excel, and SPSS version 29.

## Result and discussion

### Result

***Descriptive analysis***

Examining the descriptive analysis results, the online learning variables were generally effective, particularly in terms of student autonomy and instructor support, which consistently scored high across semesters (4.17–4.43). The active learning indicator declined slightly in semester V (3.84) but rebounded in semester VII (4.09), suggesting the need for strategies to sustain student engagement and motivation midway through the study. For the deep learning variable, deep motive and deep strategy fluctuated, decreasing in semesters III and V, then increasing again in semester VII. While the surface learning and surface motive indicators remained relatively low, indicating a tendency for students to use meaningful learning strategies. The proactive decision-making variable showed high stability across semesters, with an average score of ≥4.12 across all indicators, including goal identification, alternatives, information, decision-making, initiative, and self-improvement, reflecting stable and equitable abilities, likely influenced by the curriculum’s instilling of independence from the beginning (see Table 3).

**Table 3.** Description of semester-based online learning, deep learning, and proactive decision-making.

Variable	Indicator	Semester (M ± SD)			
		I (n = 61)	III (n = 45)	V (n = 52)	VII (n = 44)
Online learning	1. Instructor support	4.43 ± 1.01	4.17 ± 0.89	4.35 ± 0.86	4.28 ± 0.96
	2. Student interaction and collaboration	4.08 ± 1.13	3.92 ± 0.99	3.84 ± 1.12	3.99 ± 1.13
	3. Personal relevance	3.89 ± 1.22	3.81 ± 0.96	3.77 ± 1.04	4.01 ± 0.90
	4. Authentic learning	4.02 ± 1.11	3.98 ± 0.90	3.80 ± 1.07	4.00 ± 0.92
	5. Active learning	4.34 ± 0.93	4.15 ± 0.85	3.84 ± 1.10	4.09 ± 0.77
	6. Student autonomy	4.31 ± 0.98	4.17 ± 0.86	4.14 ± 0.99	4.23 ± 0.80
Deep learning	1. Deep motive	4.23 ± 1.07	3.94 ± 1.05	3.99 ± 1.10	4.09 ± 0.95
	2. Deep strategy	4.19 ± 0.93	3.90 ± 0.87	3.83 ± 1.03	4.05 ± 0.84
	3. Surface motive	3.54 ± 1.49	3.59 ± 1.29	3.73 ± 1.29	3.76 ± 1.22
	4. Surface learning	3.91 ± 1.23	3.65 ± 1.18	3.71 ± 1.22	3.88 ± 1.01
Proactive decision-making	1. Systematic identification of objectives	4.29 ± 0.65	4.18 ± 0.61	4.12 ± 0.81	4.14 ± 0.69
	2. Systematic identification of alternatives	4.34 ± 0.69	4.17 ± 0.71	4.12 ± 0.76	4.23 ± 0.69
	3. Systematic search for information	4.36 ± 0.70	4.16 ± 0.61	4.13 ± 0.79	4.23 ± 0.65
	4. Using a decision radar	4.37 ± 0.69	4.21 ± 0.55	4.07 ± 0.86	4.21 ± 0.65
	5. Taking initiative	4.32 ± 0.67	4.06 ± 0.64	4.22 ± 0.67	4.20 ± 0.67
	6. Striving for improvement	4.45 ± 0.71	4.23 ± 0.49	4.22 ± 0.67	4.27 ± 0.72

All three variables showed a positive trend from semester I to semester VII. Students rated online learning highly for instructor support and autonomy, with increased relevance of the material and authenticity of the learning in the final semester, indicating improved adaptation to the online system. The deep learning variable demonstrated dynamic academic motivation, with a decline in the middle semester but a rebound at the end of the study. While proactive decision-making remained stable with high scores across all indicators, reflecting consistent critical thinking maturity and independence. Overall, students demonstrated positive developments in independence, engagement, and reflective thinking skills throughout the learning process.

***Spearman correlation***

Spearman’s correlation results show that the authentic learning indicator in the online learning variable has the highest correlation with the deep learning approach, with ρ = 0.664 for deep motive and ρ = 0.637 for deep strategy (see Table 4). These values are in the moderate category and higher than those of the other indicators, indicating that learning that connects material to real-world contexts is closely associated with increased intrinsic motivation and the use of analytical and reflective learning strategies.

**Table 4.** Intercorrelation between online learning and deep learning.

No	Online learning	Deep learning				
		Deep motive	Deep strategy	Surface Motive	Surface strategy	Total
1	Instructor support	0.503 **	0.446 **	0.282 **	0.359 **	0.431 **
2	Student interaction and collaboration	0.391 **	0.370 **	0.307 **	0.327 **	0.391 **
3	Personal relevance	0.567 **	0.578 **	0.407 **	0.468 **	0.556 **
4	Authentic learning	0.664 **	0.637 **	0.421 **	0.497 **	0.611 **
5	Active learning	0.586 **	0.563 **	0.274 **	0.419 **	0.504 **
6	Student autonomy	0.546 **	0.580 **	0.326 **	0.476 **	0.539 **

** Correlation is significant at the 0.01 level (2-tailed).

All online learning indicators also showed a positive and significant correlation with the surface learning approach, although the strength of the relationship was lower than that for deep learning. Although online learning designs tend to support deep learning, some students still use surface learning strategies, possibly influenced by factors such as academic pressure or a focus on achieving grades.

All online learning dimensions have a positive and significant relationship with both student learning approaches, deep learning, and surface learning. However, the higher correlation with deep learning suggests that online learning designs that emphasize the authenticity of the material, personal relevance, and student autonomy are more closely associated with deep learning.

The intercorrelation between online learning and proactive decision-making indicates that the student autonomy indicator shows positive, significant correlations with all aspects of proactive decision-making, with the highest values for taking initiative (ρ = 0.429) and systematic identification of alternatives (ρ = 0.389) (see Table 5). Although in the moderate category, this finding indicates that the greater students’ autonomy in online learning, the higher their tendency to take initiative and systematically consider alternatives in decision-making.

**Table 5.** Intercorrelation between online learning and proactive decision-making.

No	Online learning	Proactive decision-making						
		Objec-tives	Alterna-tives	Infor-mation	Radar	Initia-tive	Impro-vement	Total
1	Instructor support	0.339 **	0.267 **	0.296 **	0.237 **	0.239 **	0.198 **	0.314 **
2	Student interaction and collaboration	0.335 **	0.211 **	0.258 **	0.214 **	0.291 **	0.175 *	0.295 **
3	Personal relevance	0.445 **	0.343 **	0.327 **	0.309 **	0.427 **	0.301 **	0.427 **
4	Authentic learning	0.358 **	0.292 **	0.343 **	0.321 **	0.404 **	0.297 **	0.400 **
5	Active learning	0.420 **	0.367 **	0.355 **	0.462 **	0.359 **	0.313 **	0.453 **
6	Student autonomy	0.299 **	0.307 **	0.336 **	0.389 **	0.429 **	0.230 **	0.395 **

** Correlation is significant at the 0.01 level (2-tailed).

* Correlation is significant at the 0.05 level (2-tailed).

Furthermore, the active learning and personal relevance indicators show relatively higher correlations with several aspects of proactive decision-making. For example, active learning shows the highest correlation with decision radar use (ρ = 0.462), while personal relevance shows a consistently high relationship across several indicators. It suggests that active student engagement and the material’s relevance also contribute to proactive decision-making.

Therefore, all online learning indicators show a positive and significant relationship with proactive decision-making, although the correlation strength falls in the weak to moderate category.

Overall, the Spearman correlation analysis showed a positive and significant relationship between students’ perceptions of online learning and deep learning (ρ = 0.634; p < 0.001). This coefficient value is in the moderate category, indicating that the more positive students’ perceptions of the quality of online learning—including instructor support, material relevance, active learning, and autonomy—the higher their tendency to adopt a deep learning approach, characterized by intrinsic motivation and analytical and reflective learning strategies.

Meanwhile, the correlation between online learning and proactive decision-making was also positive and significant (ρ = 0.474; p < 0.001), with the strength of the relationship in the moderate category. These findings indicate that a quality online learning experience contributes to students’ ability to make proactive decisions, including setting goals, evaluating alternatives, systematically seeking information, and demonstrating initiative in self-development (see Table 6).

**Table 6.** Spearman correlation between online learning with deep learning and proactive decision-making.

		Deep learning	Proactive decision-making
Online learning	Correlation coefficient	0.634 ^**^	0.474 ^**^
	Sig. (2-tailed)	<0.001	<0.001
	N	202	202

** Correlation is significant at the 0.01 level (2-tailed).

## Discussion

### Online learning and deep learning

The results of this study indicate a significant relationship between students’ perceptions of online learning and their tendency to use a deep learning approach, with a Spearman correlation value of ρ = 0.634. This value indicates that the more positive students’ perceptions of the quality of online learning, including instructor support, material relevance, active learning, and learning autonomy, the more likely they are to adopt a deep learning approach. This approach is characterized by intrinsic motivation to understand the material conceptually and by the use of analytical and reflective learning strategies. Therefore, the correlation result of ρ = 0.634 in this study not only indicates a strong statistical relationship but also has practical and pedagogical implications. It means that online learning designs that emphasize material authenticity, personal relevance, instructional support, and the empowerment of student autonomy will be more effective in fostering a deep learning approach. In the long term, this approach will shape students who not only master academic content but also think critically, solve problems, and possess the 21st-century competencies needed to address global challenges.

These findings align with Fullan and Langworthy’s (2014) assertion that deep learning is not merely about acquiring information but also about thinking critically, solving problems, and constructing meaning through connections to real-life contexts. Effectively designed online learning can support this by presenting authentic, flexible, and personally meaningful material to students. Furthermore, these findings corroborate those of studies by Çebi et al. (2023) and Akpen et al. (2024), which showed that online learning systems provide students with the flexibility to manage their study time and choose learning strategies that best suit their personal preferences. In this context, learning autonomy is a crucial factor in fostering higher levels of student cognitive engagement, which is at the heart of the deep learning approach. Furthermore, this study supports the findings of Amin and Shahnaz (2023) and Syawaludin et al. (2022), which emphasize the importance of project-based online learning designs for developing critical, creative, and reflective thinking skills. In project-based learning, students are challenged to solve real-world problems by applying knowledge contextually, which aligns with key indicators of deep learning.

The contribution of online learning to the development of students’ cognitive, affective, and psychomotor abilities has also been demonstrated by Musthofa et al. (2023), who showed that online learning not only influences academic achievement but also the development of professional competencies. Specifically, learning experiences that connect material to real-life situations help students understand it rather than memorize it. It is reinforced by Liu et al. (2022), who emphasized that online learning experiences encompass emotional, cognitive, and metacognitive dimensions that are essential for in-depth learning. In this context, the relationship between the quality of online learning and in-depth learning can also be understood through the Self-Determination Theory (SDT) framework developed by Ryan and Deci (2000), as adopted by Zhou et al. (2024). This theory emphasizes that when students feel autonomous, competent, and socially connected, they are more intrinsically motivated to learn deeply. Online learning that provides freedom to explore material, engage in meaningful discussions, and receive support from instructors and peers significantly supports achieving these conditions.

Although this study indicates a positive correlation between perceptions of online learning and deep learning (ρ = 0.634), learning flexibility does not automatically guarantee deep cognitive engagement. Under certain conditions, such as excessive assignment loads, time pressure, outcome-oriented assessment, and limited interaction with instructors, students may be driven to complete tasks pragmatically rather than focusing on conceptual understanding. Findings by Khong and Tanner (2024) demonstrate that a heavy course load can contribute to students’ tendency to adopt a “surface learning” approach. From the perspective of self-regulated learning, independent study requires students to plan, monitor, and evaluate their own learning processes effectively. If self-regulation skills are insufficiently developed, the autonomy inherent in such learning environments can lead to difficulties with time management, setting priorities, and maintaining consistency, thereby increasing vulnerability to academic procrastination ( Cheng & Xie, 2021; Ma et al., 2022). Therefore, to optimize deep learning, flexibility must be supported by assessments focused on understanding, reflective activities, meaningful interactions, and instructional guidance.

### Online learning and students proactive decision-making

The results of this study indicate a significant, positive relationship between online learning experiences and proactive decision-making skills, with a Spearman correlation coefficient of ρ = 0.474. It indicates that the more positive students’ experiences with online learning are, the more likely they are to make proactive decisions. It includes behaviors such as setting learning goals, systematically seeking information, evaluating alternatives, taking initiative, and actively seeking to improve themselves and their academic performance. This finding has important pedagogical implications, namely the need for educational institutions and curriculum developers to design online learning experiences that not only deliver material but also encourage the development of soft skills such as decision-making, problem-solving, and independent learning. An interactive, personalized, flexible, and reflective online learning approach is needed to develop the potential for proactive decision-making fully. In the context of online learning, students need to be encouraged to actively explore the material more broadly, read additional literature, and compare various perspectives, all of which are part of a reflective, proactive decision-making process.

Online learning is not only a medium for delivering material but also a strategic platform for developing 21st-century skills, including proactive decision-making ( Reaves, 2019). The flexible, open, and highly independent online environment encourages students to design purposeful and sustainable learning strategies ( Sumbawati et al., 2020). Within the framework of Crant’s (2000) Proactive Behavior Theory, proactive students are those who actively create positive change and take initiative in managing their learning. They leverage the flexibility of online learning to set clear academic goals, identify challenges, and design appropriate actions to achieve better results. It is also supported by a report by Sutiman et al. (2022), which found that online learning improves the quality of career decision-making by strengthening independence and fostering productive social interactions. These findings reinforce constructivist learning theory, where students do not simply receive information passively but actively construct their own knowledge by searching for, evaluating, and integrating various information sources available in the digital environment ( Bada, 2015; Taber, 2024).

The ability to identify alternative solutions, critically evaluate information, and make data-driven decisions is also influenced by access to abundant digital resources ( Tathahira, 2020). A well-designed online environment enables students to develop systematic thinking skills and make analytical decisions rather than rely on intuition. It aligns with the findings of Hongsuchon et al. (2022), which show that students in online learning systems tend to be more aware of learning strategies that align with their academic needs. Interestingly, this strong correlation reinforces the hypothesis that online learning can serve as a training tool for proactive decision-making skills, rather than simply a medium for conveying information. Students who actively engage in online learning not only complete academic assignments but also manage their learning strategically by setting goals, monitoring progress, and reflecting to support continuous improvement, all indicators of proactive decision-making skills ( Blegur, 2025; Blegur et al., 2025; Siebert et al., 2021, 2023).

This study has several limitations that should be considered when interpreting the results. First, the sample’s generalizability is limited because the respondents were drawn from a single Dance Education program, which is predominantly female (94.1%), and thus may not be representative of the broader university student population. Second, the use of a cross-sectional, correlational quantitative design that relies on self-report instruments precludes the establishment of causal relationships between variables and leaves the data susceptible to respondent perception bias. Furthermore, the descriptive analysis revealed fluctuations in motivation and “deep learning” strategies, as well as a decline in “active learning” during the intermediate semesters (semesters III and V). The underlying causes for these trends, such as assignment workloads or academic burnout, have not yet been explored through qualitative inquiry. Finally, although online learning shows a strong correlation with deep learning, the analysis also indicates a statistically significant positive correlation with “surface learning” (ρ = 0.274 to 0.497). This suggests the presence of unmeasured variables, such as evaluation systems, instructional models, or graduation requirements, that may still compel students to adopt surface-level learning strategies.

## Conclusion

Online learning designed with appropriate pedagogical principles has a positive and significant relationship with the use of deep learning approaches and students’ tendency toward proactive decision-making. These findings indicate that online learning is not simply an alternative way of delivering material but also relates to the development of reflective, independent, and adaptive student characteristics in the digital learning context. Deep learning is reflected in students’ ability to comprehensively understand concepts, think critically, and apply knowledge to real-life situations. Meanwhile, proactive decision-making relates to students’ initiative, responsibility, and ability to set goals, evaluate alternatives, and strategically manage the learning process. Amidst the transformation of higher education toward an increasingly digital and flexible system, these two aspects are becoming increasingly crucial.

The practical implications of these findings are significant for the development of digital-based higher education. Universities need to strengthen lecturers’ capacity through digital pedagogy training so they can design online learning that is interactive, relevant, and encourages active student engagement. Furthermore, e-learning systems need to be optimized with features that support discussion, reflection, progress tracking, and flexible access to materials to enhance independent learning. Curricula also need to be designed with assignments that emphasize analysis, reflection, and problem-solving to align with the development of 21st-century competencies. Overall, the results of this study provide an empirical basis for academic policy-making and strategic investment in digital learning, with a focus on strengthening the quality of student learning processes and outcomes.

## Ethics and consent

This research has obtained permission from the Research Ethics Committee of the Department of Drama, Dance, and Music, Faculty of Languages and Arts, Universitas Negeri Padang (Approval Letter No. 597b/UN35.1.5/LT/2025, dated August 10, 2025). All participants provided voluntary informed consent, both written and verbal. To protect the rights and privacy of participants, all data collected is guaranteed confidentiality and will only be used for research purposes.

## Data Availability

Open Science Framework (OSF): Quantitative data on online learning, deep learning, and students’ proactive decision-making. https://doi.org/10.17605/OSF.IO/KH97V ( Rosalina et al., 2026b). The project contains the following underlying data: Data.xlsx. Online learning, deep learning, and students’ proactive decision-making data were collected anonymously from 202 respondents. The measurement employed a five-point Likert scale. In the coding process, a score of 1 was assigned to respondents who selected *never* or *disagree*, whereas a score of 5 was assigned to those who selected *always* or *strongly agree.* Data are available under the terms of the Creative Commons Zero “No rights reserved” data waiver (CC0 1.0 Universal). Open Science Framework (OSF): Distance education learning environments survey (DELES), revised two factor study process questionnaire (R-SPQ-2F), and students proactive decision-making scale (SPDMS-18). https://doi.org/10.17605/OSF.IO/KQYAD ( Rosalina et al., 2026a). This project contains the following extended data: Distance Education Learning Environments Survey (DELES) developed by Walker and Fraser (2005). The instrument comprises 34 items across six indicators: instructor support (8 items; α = 0.87), student interaction and collaboration (6 items; α = 0.94), personal relevance (7 items; α = 0.92), authentic learning (5 items; α = 0.89), active learning (3 items; α = 0.75), and student autonomy (5 items; α = 0.75). Revised Two Factor Study Process Questionnaire (R-SPQ-2F) developed by Biggs et al. (2001). This scale (R-SPQ-2F) includes 20 items. It has been tested on 724 students using four indicators with a Comparative fit Index value of >0.90, namely deep motive of 0.997, deep strategy of 0.998, surface motive of 0.988, and surface strategy of 0.998, thus indicating good construct validity. Students’ Proactive Decision-Making Scale (SPDMS-18) developed by Blegur et al. (2025). The instrument consists of 18 items across six indicators—systematic identification of objectives (α = 0.752), systematic identification of alternatives (α = 0.765), systematic search for information (α = 0.829), use of a decision radar (α = 0.750), taking initiative (α = 0.802), and striving for improvement (α = 0.835)—with three items representing each indicator. Data are available under the terms of the Creative Commons Zero “No rights reserved” data waiver (CC0 1.0 Universal).
