# Supplementary material for: Students’ Perceptions of Online Learning and their Correlations with Deep Learning and Proactive Decision-making in Higher Education
Source: F1000Res. 2026 Oct 26;15:586. Originally published 2026 Apr 19. [Version 2] doi: 10.12688/f1000research.178825.2 (PMC13720292; doi:10.12688/f1000research.178825.2)
Supplement: Peer review history

# Reviewer report: Reviewer response for version 2

**DOI:** 10.5256/f1000research.213051.r533859

**Published:** 2026-10-29

**Authors:** Jusuf Blegur

**Affiliations:** 1 Universitas Kristen Artha Wacana, Kupang, Indonesia

Thank you for addressing my previous comments and revising the manuscript accordingly. The revisions have strengthened the discussion of several important issues related to the interpretation of the findings, methodological transparency, and contextual limitations of the study.

 I appreciate the clearer acknowledgment of the limitations associated with the sample characteristics and the generalizability of the findings. The more cautious interpretation of the relationships between students’ perceptions of online learning, deep learning approaches, and proactive decision-making is also appropriate. Given the cross-sectional correlational design, the conclusions should remain focused on associations rather than causal relationships.

 I also welcome the discussion of potential common method bias arising from the use of self-reported questionnaires, as well as the further consideration of the relationship between online learning flexibility and surface learning strategies. Addressing these issues contributes to a more balanced interpretation of the results and provides a useful basis for future research.

 Overall, the revisions have improved the clarity, rigor, and scholarly quality of the manuscript. Provided that these clarifications are adequately reflected in the final version and that the conclusions remain consistent with the study design and available evidence, I am satisfied with the revisions and recommend the manuscript for acceptance.

Is the work clearly and accurately presented and does it cite the current literature?

Yes

If applicable, is the statistical analysis and its interpretation appropriate?

Partly

Are all the source data underlying the results available to ensure full reproducibility?

Yes

Is the study design appropriate and is the work technically sound?

Yes

Are the conclusions drawn adequately supported by the results?

Partly

Are sufficient details of methods and analysis provided to allow replication by others?

Yes

Reviewer Expertise:

Physical Education Teacher Education (PETE); Physical Education Pedagogy; Instructional Design; Teacher Competence; Micro-teaching; Learning Models and Strategies; Assessment and Instrument Development

I confirm that I have read this submission and believe that I have an appropriate level of expertise to confirm that it is of an acceptable scientific standard.

# Reviewer report: Reviewer response for version 2

**DOI:** 10.5256/f1000research.213051.r533860

**Published:** 2026-10-27

**Authors:** Ananda Putriani

**Affiliations:** 1 Universitas Pendidikan Indonesia, Bandung, West Java, Indonesia

I appreciate the authors' efforts in thoroughly addressing my previous review comments. The revised manuscript demonstrates substantial improvements in methodological clarity, theoretical discussion, and the interpretation of the research findings.

 All my previous concerns have been satisfactorily addressed. I consider the revised manuscript scientifically sound and suitable for publication without further revisions.

**Review status: Approved.**

Is the work clearly and accurately presented and does it cite the current literature?

Partly

If applicable, is the statistical analysis and its interpretation appropriate?

Partly

Are all the source data underlying the results available to ensure full reproducibility?

Yes

Is the study design appropriate and is the work technically sound?

Partly

Are the conclusions drawn adequately supported by the results?

Partly

Are sufficient details of methods and analysis provided to allow replication by others?

Partly

Reviewer Expertise:

Language Education, Indonesian Language Education, Educational Research, Teaching and Learning, Higher Education

I confirm that I have read this submission and believe that I have an appropriate level of expertise to confirm that it is of an acceptable scientific standard.

# Reviewer report: Reviewer response for version 1

**DOI:** 10.5256/f1000research.197262.r526291

**Published:** 2026-10-05

**Authors:** Ananda Putriani

**Affiliations:** 1 Universitas Pendidikan Indonesia, Bandung, West Java, Indonesia

**This manuscript addresses a relevant topic and is generally well structured. However, several points require clarification and correction.**

**First, there is an inconsistency in the demographic data. Semester VII is reported to include 44 students, but the sex distribution is stated as 1 male and 53 females, which totals 54. Please verify and correct these figures.**

**Second, the reported age statistics are inconsistent between the Abstract and Methods sections. The Abstract reports M = 20.46, SD = 1.75, whereas the Methods section reports M = 20.2, SD = 1.9. Please report these values consistently.**

**Third, the normality test results should be checked. The Shapiro–Wilk p-value for online learning differs between the narrative and Table 1.**

**Fourth, please clarify the term “multiple correlation design,” since the reported analyses mainly involve separate Spearman correlations.**

**Finally, the manuscript should use correlational rather than causal language. Because the study is correlational, terms such as “impact” should be replaced with “association” or “relationship” where appropriate.**

**Overall, the study is relevant and potentially valuable, but minor revisions are needed to improve consistency and methodological clarity.**

Is the work clearly and accurately presented and does it cite the current literature?

Partly

If applicable, is the statistical analysis and its interpretation appropriate?

Partly

Are all the source data underlying the results available to ensure full reproducibility?

Yes

Is the study design appropriate and is the work technically sound?

Partly

Are the conclusions drawn adequately supported by the results?

Partly

Are sufficient details of methods and analysis provided to allow replication by others?

Partly

Reviewer Expertise:

Language Education, Indonesian Language Education, Educational Research, Teaching and Learning, Higher Education

I confirm that I have read this submission and believe that I have an appropriate level of expertise to confirm that it is of an acceptable scientific standard, however I have significant reservations, as outlined above.

# Response

**Published:** 2026-10-17

**Authors:** Venny Rosalina

**Affiliations:** Seni Drama Tari dan Musik, Universitas Negeri Padang, Padang, West Sumatra, Indonesia

We would like to express our sincere gratitude to the reviewer for the thorough, constructive, and helpful feedback regarding our manuscript, "Students’ perceptions of online learning and their correlations with deep learning and proactive decision-making in higher education." We have carefully addressed each point raised and made the necessary corrections to ensure numerical consistency, methodological clarity, and precise linguistic phrasing throughout the manuscript.

 Regarding the first point on demographic data, we have thoroughly audited and verified the respondent figures. The distribution for Semester VII has been corrected to 54 students (1 male and 53 females, representing 21.8% of the total sample), resolving a previous typographical inconsistency and ensuring that the semester breakdown correctly totals 202 participants across all cohorts.

 In response to the second point concerning age statistics, we have synchronized all reported values across the Abstract and Methods sections. The age parameters are now consistently presented as M±SD=20.46±1.75 years across all sections of the paper.

 Third, regarding the normality test results, we re-examined the statistical output and aligned the narrative description precisely with Table 1. The narrative now explicitly clarifies that, although the online learning variable appeared normally distributed in the Kolmogorov–Smirnov test (p=0.069), the Shapiro–Wilk test indicated a significant departure from normality (p<0.001). Combined with the non-normal distribution observed for both deep learning and proactive decision-making (p<0.05 across both tests), this justified the application of non-parametric procedures.

 To address the fourth comment, we refined our description of the research design. We replaced ambiguous terms such as "multiple correlation design" with a clear statement that a correlational research design using Spearman’s rank-order correlation was implemented to analyze the bivariate associations between the predictor variable (online learning) and each outcome variable (deep learning and proactive decision-making) individually.

 Finally, we conducted a systematic textual review to eliminate any phrasing that might imply direct causality. All instances of causal terms, including "impact" and "effect," have been replaced with appropriate relational terms, such as "association," "relationship," or "correlation," to accurately reflect the study's correlational nature.

# Reviewer report: Reviewer response for version 1

**DOI:** 10.5256/f1000research.197262.r511756

**Published:** 2026-09-07

**Authors:** Jusuf Blegur

**Affiliations:** 1 Universitas Kristen Artha Wacana, Kupang, Indonesia

This study, entitled *“Online Learning, Deep Learning, and Students’ Proactive Decision-Making,”* examines the relationships between students’ perceptions of online learning, deep learning approaches, and proactive decision-making competencies. The study involved 202 undergraduate students from the Dance Education Study Program at Universitas Negeri Padang, West Sumatra, Indonesia. The topic is timely and relevant, particularly given the growing importance of online learning environments in higher education and the increasing need to develop students’ autonomy, active engagement, and decision-making capacities.

 The findings indicate that students’ perceptions of online learning are significantly and positively associated with their deep learning approaches. The relationship is moderate to strong (ρ = 0.634, p < 0.001), suggesting that students who perceive online learning more positively tend to demonstrate stronger tendencies toward deep learning. In particular, authentic learning and student autonomy appear to be the most prominent indicators associated with this relationship. These findings suggest that online learning may provide meaningful opportunities for students to engage with learning materials more independently and authentically, thereby supporting deeper approaches to learning.

 The study also demonstrates a significant positive relationship between perceptions of online learning and proactive decision-making (ρ = 0.474, p < 0.001). Although the magnitude of this relationship is moderate, the finding indicates that students who perceive their online learning experiences more positively also tend to demonstrate stronger proactive decision-making competencies. Active learning and student autonomy appear to have particularly important associations with students’ abilities to evaluate alternatives, take initiative, and make decisions independently. From a pedagogical perspective, these findings position online learning not merely as a mechanism for delivering instructional content but also as a potential learning environment for fostering autonomy, active participation, intrinsic motivation, and self-regulated decision-making.

 Methodologically, the study demonstrates several strengths. The literature review is relatively current and incorporates recent studies published between 2024 and 2026 while maintaining connections with established theoretical perspectives, including Self-Determination Theory and Proactive Behavior Theory. The use of validated instruments, including the DELES, R-SPQ-2F, and SPDMS-18, further strengthens the methodological foundation of the study. The researchers also provide sufficient information concerning the population, sample characteristics, measurement dimensions, and statistical procedures, including the use of SPSS version 29, which contributes to the replicability of the study. The application of Spearman’s rank-order correlation is appropriate given the non-normal distribution of the data identified through the normality tests. Overall, the statistical procedures are consistent with the correlational nature of the research questions.

 Nevertheless, several aspects of the manuscript would benefit from further clarification and refinement.

 First, the authors should explicitly acknowledge the limitations concerning the generalizability of the findings. The sample was drawn from a single Dance Education Study Program, and the sample was highly dominated by female students (94.1%). Consequently, the findings should be interpreted within the specific institutional, disciplinary, and demographic context of the participants rather than generalized broadly to undergraduate students in higher education.

 Second, the manuscript should briefly address the potential issue of common method bias because the principal constructs were measured using cross-sectional, self-reported questionnaires. The authors should clarify what procedural safeguards were implemented during data collection to minimize potential response bias and common method effects. Even a concise explanation would strengthen the methodological transparency of the study.

 Third, the interpretation of the findings should be carefully aligned with the correlational design. The observed correlations demonstrate statistically significant associations but do not establish causal relationships. Therefore, expressions such as “impacts,” “causes,” or “drives” should be replaced with more appropriate correlational terminology, such as “is associated with,” “is positively related to,” or “correlates with.” Any causal interpretation would require stronger evidence from an experimental, quasi-experimental, or longitudinal design.

 Finally, the discussion would be substantially strengthened by providing a deeper explanation of the finding that online learning flexibility may, under certain circumstances, be associated with surface learning strategies. This issue deserves more than a brief mention in the Results section. The authors could consider whether assignment workload, assessment practices, time pressure, limited instructor interaction, or predominantly task-oriented learning activities may encourage students to adopt surface approaches despite the flexibility provided by online learning. Theoretical perspectives on student autonomy and self-regulated learning could also be used to explain why flexibility does not necessarily lead to deeper engagement. Addressing this apparently contradictory finding would make the discussion more critical, balanced, and theoretically meaningful.

 Overall, the manuscript presents a relevant and empirically supported contribution to the discussion of online learning in higher education. The statistical findings are clear, the methodological approach is generally appropriate, and the practical implications are potentially valuable. The manuscript appears suitable for further consideration following minor revision, particularly to strengthen the discussion of limitations, address common method bias, moderate causal language, and provide a more nuanced theoretical interpretation of the relationship between online learning flexibility and surface learning strategies.

Is the work clearly and accurately presented and does it cite the current literature?

Yes

If applicable, is the statistical analysis and its interpretation appropriate?

Partly

Are all the source data underlying the results available to ensure full reproducibility?

Yes

Is the study design appropriate and is the work technically sound?

Yes

Are the conclusions drawn adequately supported by the results?

Partly

Are sufficient details of methods and analysis provided to allow replication by others?

Yes

Reviewer Expertise:

Physical Education Teacher Education (PETE); Physical Education Pedagogy; Instructional Design; Teacher Competence; Micro-teaching; Learning Models and Strategies; Assessment and Instrument Development

I confirm that I have read this submission and believe that I have an appropriate level of expertise to confirm that it is of an acceptable scientific standard, however I have significant reservations, as outlined above.

# Response

**Published:** 2026-10-17

**Authors:** Venny Rosalina

**Affiliations:** Seni Drama Tari dan Musik, Universitas Negeri Padang, Padang, West Sumatra, Indonesia

We would like to express our sincere gratitude to the reviewer for the highly insightful, constructive, and encouraging feedback on our manuscript, "Students’ perceptions of online learning and their correlations with deep learning and proactive decision-making in higher education." We have carefully addressed each point raised and incorporated substantial revisions to enhance the paper's methodological clarity, linguistic precision, and theoretical depth.

 Regarding sample generalizability, we fully concur with the reviewer's observation. In the revised manuscript, we have explicitly addressed this constraint within the Limitations section, noting that our participants were drawn exclusively from a single Dance Education program and were predominantly female (94.1%). Consequently, we have clarified that our findings should be interpreted strictly within this specific institutional, academic, and demographic context rather than generalized to the broader undergraduate population.

 To address concerns regarding potential common method bias and cross-sectional constraints, we have expanded our methodological reflections. We explicitly acknowledge that relying on cross-sectional, self-reported questionnaires leaves the data susceptible to respondent perception bias and common method variance. We have elaborated on the procedural safeguards implemented during data collection—such as assuring respondent anonymity and emphasizing honest reporting—to mitigate these risks, while recommending that future studies incorporate longitudinal or qualitative inquiries to capture complex learning trends over time.

 Furthermore, we conducted a thorough textual review to align all interpretations strictly with a correlational research design. Any terminology implying direct causality (such as "impacts," "causes," or "drives") has been systematically replaced with precise relational phrasing, including "is associated with," "correlates positively with," and "is linked to." We re-emphasized that while Spearman's rank correlation evaluates the strength and direction of these associations, it does not establish causal directionality.

 Finally, we significantly enriched the Discussion section to provide a nuanced theoretical explanation for the observed link between online learning flexibility and surface learning strategies. Drawing upon Self-Regulated Learning (SRL) theory, we discuss how learning autonomy does not automatically guarantee deep cognitive engagement. Without well-developed self-regulation skills, students facing heavy course loads, strict time constraints, and outcome-oriented assessments may struggle with time management and default to pragmatic, surface-level approaches (Khong & Tanner, 2024; Cheng & Xie, 2021; Ma et al., 2022). Furthermore, we contextualize the positive correlation with surface learning (ρ = 0.274 to 0.497) as an indicator of unmeasured structural variables—such as evaluation frameworks or graduation requirements—that continue to influence student learning behaviors.
